# On variability in local field potentials

**DOI:** 10.1038/s42003-026-10881-x

**Published:** 2026-09-02

**Authors:** Mohsen Parto-Dezfouli, Elizabeth L. Johnson, Eleni Psarou, Conrado Arturo Bosman, B. Suresh Krishna, Pascal Fries

**Affiliations:** 1 Max Planck Institute for Biological Cybernetics, Tübingen, Germany; 2 Ernst Strüngmann Institute (ESI) for Neuroscience in Cooperation with Max Planck Society, Frankfurt, Germany; 3 Goethe University Frankfurt, University Hospital, Department for Psychiatry, Psychosomatic Medicine and Psychotherapy, Frankfurt, Germany; 4 Goethe University Frankfurt, Cooperative Brain Imaging Center - CoBIC, Frankfurt, Germany, Frankfurt, Germany; 5 Departments of Medical Social Sciences and Pediatrics, Northwestern University, Chicago, IL USA; 6 Department of Psychology, Northwestern University, Evanston, IL USA; 7 Cognitive and Systems Neuroscience Group, Swammerdam Institute for Life Sciences, University of Amsterdam, Amsterdam, The Netherlands; 8 Department of Physiology, McGill University, Montreal, QC Canada

**Keywords:** Neural encoding, Neural decoding, Sensory processing

## Abstract

Neuronal variability is a fundamental feature of neuronal coding. In spiking activity, across-trial variance (ATV) normalized by mean spike count (i.e., the Fano factor) shows stimulus-induced quenching. ATV has also been studied in electro/magneto-encephalography (E/MEG) without normalization, revealing effects of stimulation and cognition. Here we show that outside of event-related potentials (ERPs), ATV for both EEG and local field potential (LFP) is nearly identical to intra-trial variance (ITV), which equals signal power. ATV–ITV correlation decreases during ERPs, proportional to how much the ERP explains total power. While EEG ATV shows post-stimulus quenching, LFP ATV does not, particularly in the gamma band, where power increases after stimulus onset. To provide an independent variability measure, we introduce CV(power), the coefficient of variation of signal power, as a mean-normalized metric. CV(power) is the inverse of the signal-to-noise ratio of power and thus related to decodability, offering a variability measure applicable to E/MEG and LFP.

## Abstract (web summary)

Parto-Dezfouli et al. analyze variability in LFP and EEG signals. They find that the previously used across-trial variability is almost identical to within-trial variability, i.e., the usual power metric, while the coefficient of variation of power better captures variability.

## Introduction

The presence and the properties of a visual stimulus can be decoded from the spike counts (SCs) of neurons in the early visual cortex. These SCs can be considered a signal in the context of signal detection theory^1^. In this theory, the signal emitted by a given neuron in response to a given stimulus is ideally identical for each presentation of that stimulus, i.e., there is no noise or variability across trials. However, empirically, different trials of presentation of a given stimulus lead to SCs that vary, i.e., there is across-trial variability (ATV). ATV of SCs has been found in many brain areas of many species^2–8^. In order to investigate SC ATV further, it needs to be properly normalized by the mean SC, because changes in the mean are expected to trivially lead to corresponding changes in the ATV. Specifically, neuronal spiking has often been modeled as a Poisson process with a spike rate that depends, e.g., on stimulus conditions^9^. For a Poisson or quasi-Poisson process, the SC variance scales linearly with the SC mean, and their ratio is constant (at a value of one for a Poisson process). Thus, a stimulus-related change in SC variance might be simply due to a corresponding change in SC mean. By contrast, a stimulus-related change in the ratio of SC variance over SC mean, i.e., in the Fano Factor, would demonstrate a stimulus-related change in the mean-normalized variance of the SC code. It is a milestone finding in neuroscience that the SC Fano Factor is reduced by stimulus onset, leading to the conclusion that stimulus onset quenches neuronal variability^4^. In addition to sensory stimuli, non-sensory factors such as arousal, attention, and adaptation can also reduce neuronal variability^7,10^. Identifying such changes in variance (independent of the mean) has implications for decoding and has led to actionable constraints on neural-network models and increased our understanding of the underlying mechanisms^11–14^.

More recently, ATV has been investigated for continuous signals, and several studies using electro/magnetoencephalography (E/MEG) have shown reductions in ATV following a stimulus and with attention^15–20^. These studies have typically calculated the variance of the raw or band-pass filtered EEG- or MEG-signals across trials. Those studies also reported that ATV often includes information that is masked by traditional event-related potential (ERP) analysis^21^. Here, using three datasets (two LFP and one EEG dataset), we show that outside of ERPs, the LFP and EEG variance calculated across trials (the ATV) and subsequently averaged within a given time window is highly correlated with, and in fact almost identical to, the variance calculated within that time window (the ITV, for intra-trial variability) and subsequently averaged over trials. Since ITV defined in this manner is exactly the same as signal power, the ATV calculated in prior studies outside of ERPs is not an independent measure of variability, but is instead almost identical to signal power, which is a well-established measure. The high correlation and near-identity between ATV and ITV are reduced during ERPs, and the degree to which the ERP explains total power is correlated to the reduction in ATV-ITV correlation. The near-identity of ATV and power (ITV) can in fact be expected from mean-field neuronal-population models that model the LFP as a result of a stochastically driven near-linear system^22–24^. In such systems, the variance across multiple realizations of the process is expected to correspond to the variance across time within one realization. This property is called ergodicity. Even though the LFP and EEG signals are often not fully stationary within the trial (and thus deviate from ergodicity), the signal power (ITV) remains almost identical to the ATV, except for the ERP period. We demonstrate by simulation that ATV and ITV are indeed expected to be identical for ongoing activity, and that the addition of a constant component across trials, i.e., the ERP, breaks this equivalence^25,26^.

Analyzing our three datasets, we confirm that ATV for EEG shows a clear post-stimulus quenching, as shown in prior studies^15–20^, but the ATV for LFP does not consistently show post-stimulus quenching. The only prior study of ATV in LFP data that we know of reported a post-stimulus increase in ATV^27^. Our results here are also consistent with those of prior studies that showed a high correlation between ATV and signal power in different frequency bands below 40 Hz^18,19^. We substantially extend these results by showing that these measures are in fact almost identical (at least in our data).

Thus, our results challenge the common interpretation of ATV reductions in continuous signals as reflecting variability quenching analogous to that observed in spike-count measures. Instead, we show that ATV in EEG and LFP signals closely tracks signal power, consistent with predictions from mean-field and population-level models, in which field potentials arise from stochastically driven, near-linear systems^22–24^. This reinterpretation has implications for frameworks such as predictive coding and population coding models, where reductions in variability are often linked to improved encoding efficiency or reduced uncertainty. Our findings suggest that, for continuous signals, apparent variability reductions may instead reflect changes in signal power rather than intrinsic noise suppression, thereby requiring a more careful distinction between power modulation and true variability quenching.

A key motivation for this study was to provide a measure of variability independent of signal power, i.e., a meaningful variability metric that removes the influence of the mean power. Following the same heuristic motivation as in the Fano factor, we introduce the coefficient of variation (CV) of signal power as a mean-normalized metric of variability for continuous neuronal data. The CV(power), defined as the standard deviation of power across trials divided by the mean power across trials, controls for changes in power variability that are directly proportional to changes in mean power. We will show that CV(power) is not affected by scaling of the signal. Because the SD is the square root of variance, and variance scales with the squared signal, the SD scales proportionally with the mean. Consequently, the CV(power) remains constant under amplitude scaling, highlighting its suitability as a power-independent measure of variability.

The CV is a well-established statistical measure previously used in other neuroscientific contexts^28,29^. Here, we introduce and validate the use of CV(power) as a principled, mean-normalized measure of variability in continuous neuronal data. We demonstrate that the CV(power) metric shows clear post-stimulus quenching for lower-frequency bands in most cases for both LFP and EEG signals in our datasets.

## Results

### Overview

We analyzed three empirical datasets: macaque electrocorticography (ECoG), macaque intracortical microelectrode recordings, and human EEG, as well as one simulated dataset. The macaque ECoG recordings, which exhibit clear modulation of gamma and alpha-beta oscillations, served as a gold-standard meso-scale dataset for examining how oscillatory components influence ATV. Simulations were used to interpret empirical findings and to illustrate that ATV is identical to power (ITV) under ergodic conditions, and also the independence of the CV(power) metric from mean power. Intracortical microelectrode recordings, including both single-unit activity (SUA) and LFP, allowed comparisons between the classical single-unit spike-count-derived Fano factor, the LFP-derived ATV, and our proposed CV(power) metric. Finally, the human EEG data enabled us to extend our findings to the EEG domain, in which ATV has been most often studied, and to assess the generality of our observations across modalities and species; furthermore, the larger number of subjects in the EEG study allowed us to test this metric on a population of subjects.

**Fig. 1:**
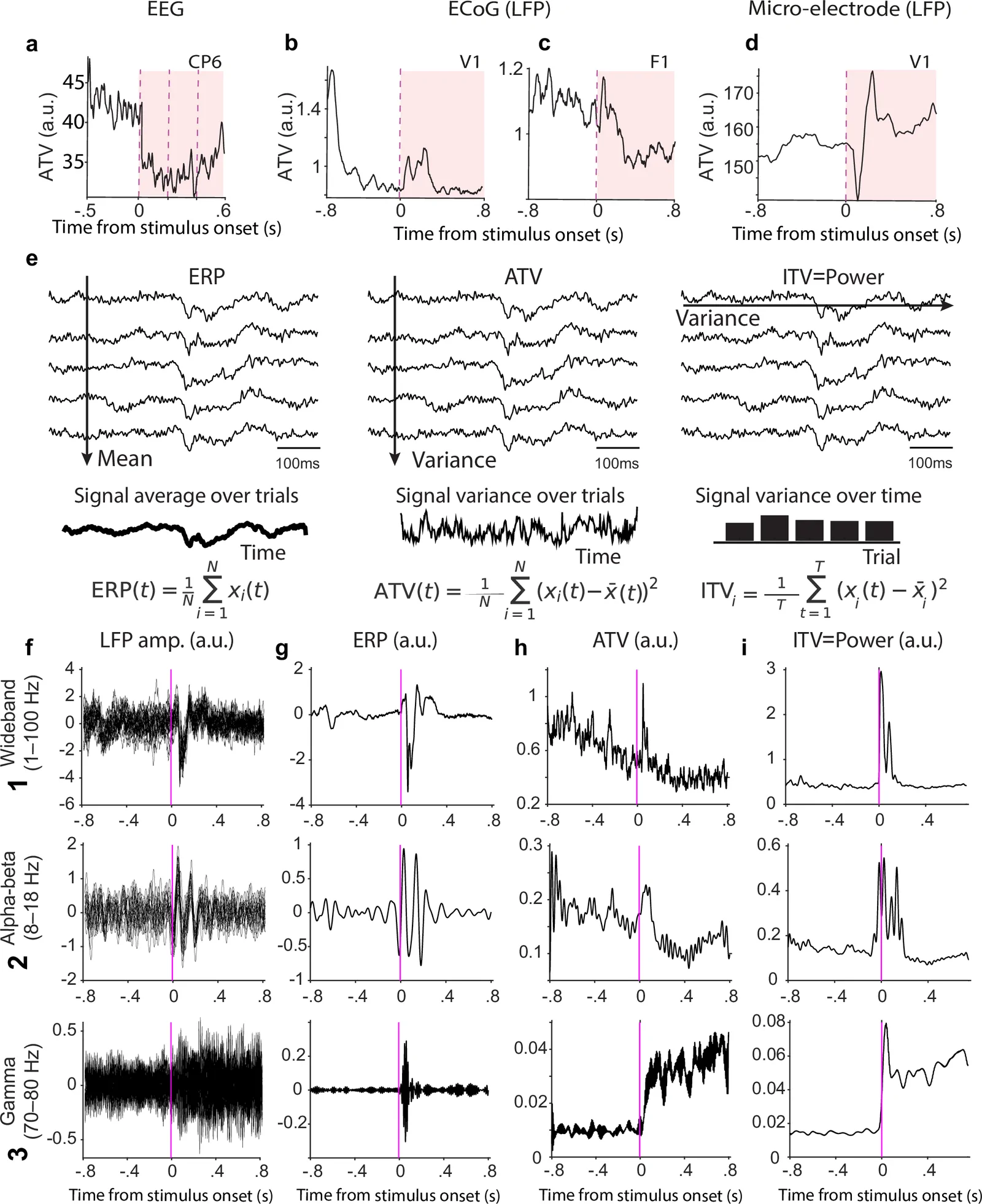
ERP, ATV, ITV (power): concepts and empirical examples. **a** Wideband ATV calculated on data from EEG channel CP6 from one example human participant, during fixation and stimulus presentation. **b** Same as (**a**), but averaged over all ECoG electrodes on area V1 in monkey K. **c** Same as (**b**), but for electrodes on area F1. **d** Same as (**b**), but averaged over multiple contacts and sessions of laminar multi-contact recordings in area V1 of monkey E. Shaded areas indicate the stimulus period. **e** Schematic illustration of event-related potential (ERP), across-trial variability (ATV), and intra-trial variability (ITV) or power (i: trial number from 1 to N; t: time point from 1 to T). LFPs (**f**), ERPs (**g**), ATV (**h**), and ITV (**i**), separately for wideband data (row 1, see label to the left), data filtered in the alpha-beta frequency band (8–18 Hz; row 2), and the gamma frequency (70–80 Hz; row **3**). The LFPs are from 150 example trials recorded from one example ECoG channel in area V1 of Monkey K during fixation and stimulus presentation. The ITV (i.e., power) in (**i**) was calculated with sliding windows of 0.05 s length, which causes a temporal smearing such that ITV increases already at or slightly before stimulus onset. LFP signals have been *z*-scored, such that the LFP and derived metrics are reported in arbitrary units (a.u.).

First, we followed the same approach as used in previous E/MEG ATV studies and calculated the ATV of the raw EEG or LFP. We found an ATV decrease after stimulus presentation for an EEG dataset, as in prior reports (Fig. 1a), but also found a wider variety of ATV changes after stimulus presentation in two LFP datasets based on ECoG and micro-electrode recordings, respectively (Fig. 1b–d). A schematic overview of the three key metrics used in this study, namely ERP, ATV, and ITV (power), is provided in Fig. 1e. This illustration depicts how each measure is derived from the same set of trials: the ERP as the average over trials, the ATV as the variance across trials, and the ITV or power as the variance across time points within trials. Note that spectrally resolved power is commonly calculated as the average over the squared Fourier transforms of the individual trials^30–32^. Yet it is important to note that the power per frequency band is equivalent to the ITV of the signal after filtering for this frequency band. Correspondingly, the ITV of the wideband signal corresponds to the power summed over all frequencies.

### ATV resembles power

In our ECoG dataset, we calculated the ATV of the LFP recorded from electrodes covering 15 brain areas of an awake macaque monkey (monkey K, same dataset as shown in Fig. 1b, c). During the experiment, the monkey kept its gaze on the fixation point while covertly attending to one of two simultaneously presented stimuli. The attention task resulted in two conditions (see “Methods” for details): (1) a condition with attention to the hemifield contralateral to the recorded brain areas, referred to as the attend IN condition (as most visually responsive brain areas are more sensitive for stimuli in their contralateral hemifield), (2) a condition with attention to the ipsilateral hemifield, referred to as attend OUT condition.

We considered 150 example trials of LFP recordings from one electrode on area V1 of Monkey K, both wideband (Fig. 1f1) and after bandpass filtering in the alpha-beta band (8–18 Hz, Fig. 1f2) or the gamma band (70–80 Hz, Fig. 1f3). We calculated the ERP time courses (Fig. 1g), the corresponding ATV time courses (Fig. 1h), and the ITV (Fig. 1i) within 0.05 s windows moved in 1 ms steps. The ATV of the wideband LFP exhibited a transient increase after stimulus onset, which was superimposed on a slower overall decrease across the analyzed task period. A similar pattern could be seen in the ATV of the alpha-beta filtered LFP (Fig. 1h2). Notably, the ATV of the gamma-filtered LFP showed a stimulus-related increase that was sustained for the duration of stimulation (Fig. 1h3). Crucially, these changes in ATV were similar to the changes in the corresponding ITV values (Fig. 1i). As mentioned above, ITV for bandpass-filtered signals is equivalent to the power in the respective bands.

**Fig. 2:**
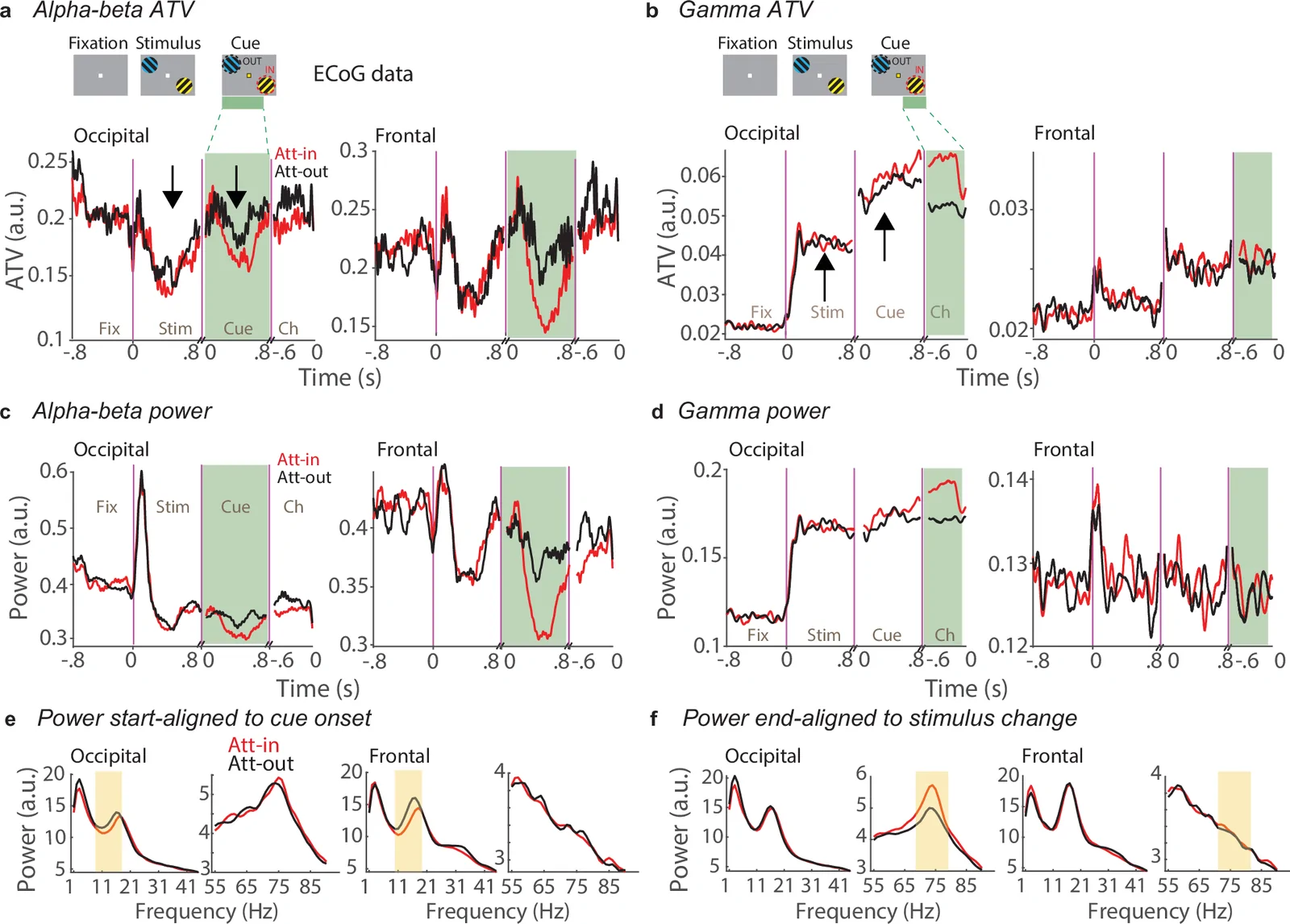
ATV and power. **a** Time-course of average ATV of the LFP filtered in the alpha-beta band over occipital (left panel) and frontal (right panel) clusters for attend-IN (red) and attend-OUT (black) conditions, in ECoG data of Monkey K. Arrows indicate the reduction of ATV during stimulus presentation and attention periods. Results were smoothed using a 0.02 s sliding window. *Fix*: fixation, S*tim*: stimulus, *Cue*: aligned to attention cue onset, *Ch*: aligned to stimulus change. As the data were *z*-scored prior to variance estimation, ATV is given in arbitrary units (a.u.). Green shaded areas were used for power analysis in (**e**). **b** Same as (**a**), but for the LFP filtered in the gamma band. Green shaded areas were used for power analysis in (**f**). **c** Time-course of averaged power of the LFP filtered in the alpha-beta band over occipital (left panel) and frontal (right panel) clusters for attend-IN (red) and attend-OUT (black) conditions. Here, power was calculated as the square of the absolute (rectified) value of the LFP. Results were smoothed using a 0.02 s sliding window. **d** Same as (**c**), but for the gamma band. **e** LFP power spectra for the period 0.3–0.8 s after cue onset, separately for attend-IN (red) vs. attend-OUT (black) conditions. The yellow shaded area indicates the alpha-beta band used in (**a**, **c**). **f** Same as (**e**), but for the period 0.5 s before the stimulus change. The yellow shaded area indicates the gamma band used in (**b**, **d**).

To further investigate the relationship between ITV, i.e., power, and ATV, we focused on two clusters of areas in the same ECoG dataset based on their dominant peak power, as we have established previously^33^: an occipital cluster containing areas V1, V2, and V4, and a frontal cluster containing areas F1, F2, and F4. In these two clusters of areas, we calculated ATV in the alpha-beta and gamma bands, respectively. ATV was computed for each time point across four periods of the task: fixation (last 0.8 s before stimulus onset), stimulus onset (0.8 s following stimulus onset), attention-cue onset (0.8 s following cue onset), and sustained attention (last 0.6 s before stimulus change). ATV in the alpha-beta band (8–18 Hz) showed decreases after the stimulus onset and after the cue onset (Fig. 2a; two-sided Wilcoxon rank-sum test, occipital: *p*_stimulus_ = 7.87 × 10^−7^, *p*_cue_ = 1.83 × 10^−9^, frontal: *p*_stimulus_ = 1.89 × 10^−3^, *p*_cue_ = 2.78 × 10^−10^). The decrease after the cue onset appeared stronger for attend-IN versus attend-OUT, in particular for the frontal areas (Fig. 2a red versus black lines, two-sided Wilcoxon rank-sum test, occipital: *p*_IN-OUT_ = 1.89 × 10^−2^, frontal: *p*_IN-OUT_ = 2.62 × 10^−5^). In the occipital cluster, ATV in the gamma band (70–80 Hz) showed a first increase after stimulus onset and a further increase after cue onset (Fig. 2b; two-sided Wilcoxon rank-sum test, occipital: *p*_stimulus_ = 6.65 × 10^−11^, *p*_cue_ = 1.43 × 10^−11^, frontal: *p*_stimulus_ = 0.358, *p*_cue_ = 0.302). In the occipital cluster, the increase after cue onset appeared stronger for the attend-IN than the attend-OUT condition, and this difference was further accentuated during the last 0.6 s before the stimulus change (Fig. 2b red versus black lines; two-sided Wilcoxon rank-sum test, occipital: *p*_IN-OUT_ = 9.09 × 10^−5^, frontal: *p*_IN-OUT_ = 0.821).

Intriguingly, these effects of stimulus and attention on the ATV of the bandpass-filtered LFPs are closely recapitulated in the power calculated as the squared absolute value of the bandpass-filtered LFPs (Fig. 2c, d): Power in the alpha-beta band decreased after the stimulus onset and after the cue onset (two-sided Wilcoxon rank-sum test, occipital: *p*_stimulus_ = 3.86 × 10^−7^, *p*_cue_ = 2.24 × 10^−8^, frontal: *p*_stimulus_ = 7.01 × 10^−4^, *p*_cue_ = 1.22 × 10^−8^). The decrease after the cue onset appeared stronger for attend-IN versus attend-OUT, in particular for the frontal areas (two-sided Wilcoxon rank-sum test, occipital: *p*_IN-OUT_ = 0.127, frontal: *p*_IN-OUT_ = 2.33 × 10^−4^) (Fig. 2c). Also, power in the gamma band showed a first increase after stimulus onset and a further increase after cue (two-sided Wilcoxon rank-sum test, occipital: *p*_stimulus_ = 6.30 × 10^−11^, *p*_cue_ = 2.79 × 10^−11^, frontal: *p*_stimulus_ = 0.269, *p*_cue_ = 0.135). In the occipital areas, the increase in the last 0.6 s before the stimulus change appeared stronger for the attend-IN than the attend-OUT condition (two-sided Wilcoxon rank-sum test, occipital: *p*_IN-OUT_ = 1.11 × 10^−3^, frontal: *p*_IN-OUT_ = 0.60) (Fig. 2d).

Moreover, the corresponding power spectra reveal similar reductions of the alpha-beta power (Fig. 2e; two-sided Wilcoxon rank-sum test, occipital: *p*_alpha_ = 8.72 × 10^−5^, *p*_gamma_ = 0.860, frontal: *p*_alpha_ = 2.50 × 10^−10^, *p*_gamma_ = 0.772) and increases of the gamma power (Fig. 2f; two-sided Wilcoxon rank-sum test, occipital: *p*_alpha_ = 0.177, *p*_gamma_ = 2.83 × 10^−9^, frontal: *p*_alpha_ = 0.374, *p*_gamma_ = 0.459) for the attend-IN compared to the attend-OUT condition^34^.

### ATV is highly correlated with ITV, which corresponds to power

These results suggested that ATV might be related to power, in line with previous results showing an ATV-power relationship based on power spectra^18,19,35^. While ATV is calculated across trials per time point, power is calculated across time points per trial, typically followed by averaging over trials. We further investigated this putative relation between ATV and power. The power of a signal corresponds to the variance of the signal across time, defined as the sum of the squared deviations from the signal mean. Thus, signal power corresponds to the ITV, and we use this acronym because it exposes the similarities and differences between ATV and ITV: ATV quantifies the variability across trials per time point, and can subsequently be averaged over time points; ITV quantifies the variability across time points per trial, and can subsequently be averaged over trials. If the signal has been filtered, ITV corresponds to the power in the corresponding frequency band; if the signal has not been filtered, ITV corresponds to the total power of the signal.

**Fig. 3:**
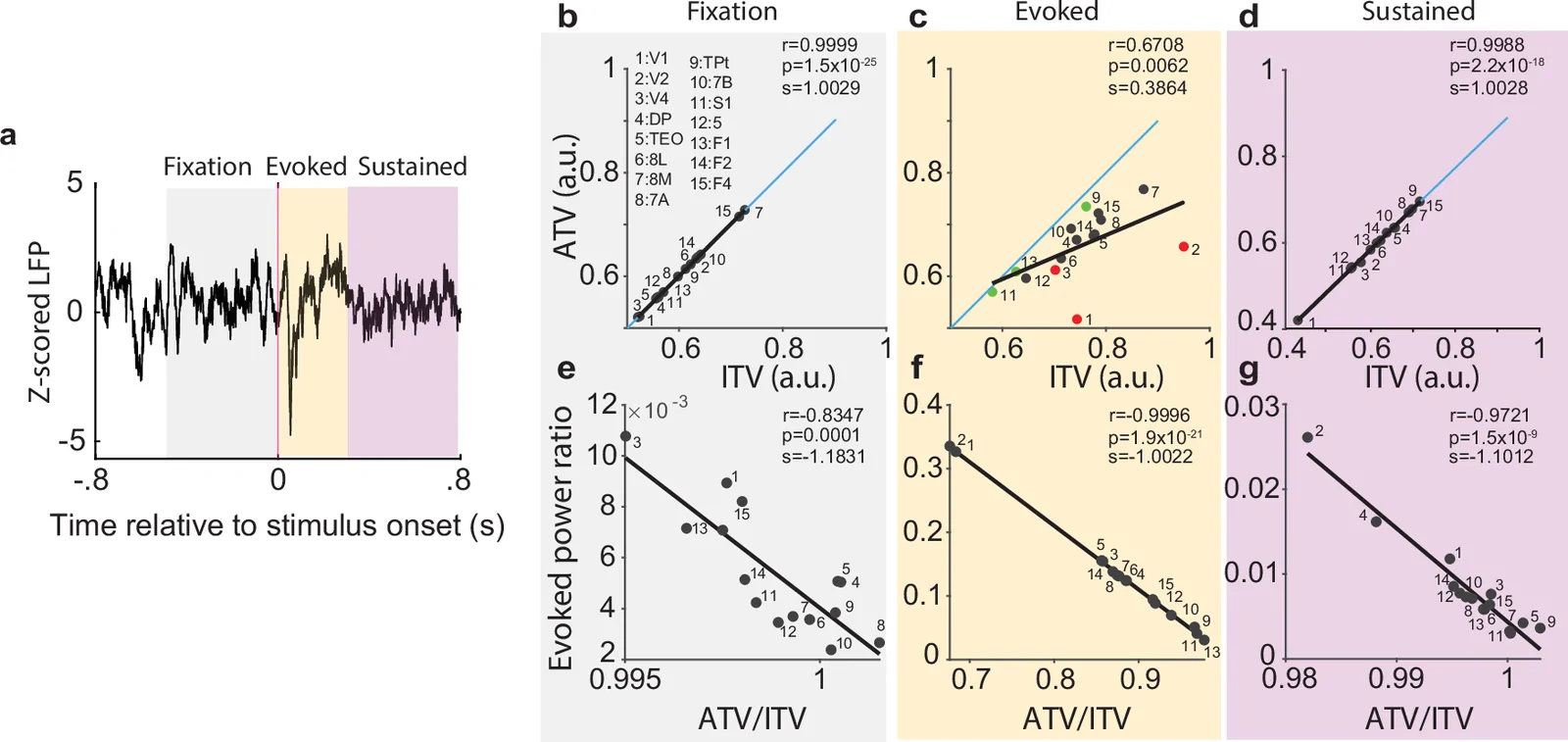
ATV correlates with ITV. **a** Averaged z-scored LFP for three task periods: Fixation (gray), evoked-response period (yellow), and sustained-activity period (purple). **b** Scatter plots of ATV versus ITV across the 15 indicated brain areas for the Fixation period. The values in the upper right corner provide the Pearson correlation coefficient (*r*), its significance level (*p*), and the slope of a linear regression (*s*, shown as a black line) between ATV and ITV across the 15 areas. The blue diagonal line indicates the line of equality between ATV and ITV. As the data were *z*-normalized prior to variance estimation, both ATV and ITV are in arbitrary units (a.u.). **c** Same as (**b**), but for the evoked-response period. **d** Same as (**b**), but for the sustained-activity period. **e–g** Correlation between the evoked power ratio, that is, the ratio of the evoked power over the total power, and the ratio of ATV over ITV, separately for each task period. The values in the upper right corners of each panel show the same analyses as in (**b**).

For the following direct comparison between ITV and ATV, we opted to use the unfiltered wideband signal (only including hardware filters during data acquisition, but no offline filtering), because it has been used for most applications of ATV on continuous neuronal signals in the literature. While ATV has typically been calculated separately per time point, this is not possible for ITV. Rather, the calculation of ITV, both for the unfiltered and the filtered signal, requires multiple samples per trial and thereby some time period. Therefore, to directly compare ITV and ATV for the same data, we defined three periods: (1) the fixation period (last 0.5 s before stimulus onset), (2) the evoked response period (first 0.3 s following stimulus onset), and (3) the sustained-response period (0.3 to 0.8 s after stimulus onset). ITV was calculated per trial and per period across the time points, and then averaged over trials. ATV was calculated across trials per time point, and then averaged over time points (see Fig. 1e for a schematic illustration). We used the same ECoG data as reported above. Both ITV and ATV were calculated for individual recording channels and subsequently averaged over channels within each recording area, separately for the three periods as indicated by the colored backgrounds in Fig. 3a. Figure 3b–d shows scatter plots of ATV versus ITV across 15 brain areas, separately for the aforementioned periods of fixation (Fig. 3b), evoked response (Fig. 3c), and sustained-response (Fig. 3d). Each scatter plot shows the diagonal as a blue line, with the points on/above/below the diagonal showing that ATV is equal to/larger/smaller than ITV. In addition, each plot shows the linear regression of ATV versus ITV as a black line. This reveals a significant positive correlation between ATV and ITV in all three periods. Notably, the regression coefficient between ATV and ITV assumes values of 0.9999 (Fig. 3b) and 0.9988 (Fig. 3d) during the fixation and sustained-response periods, respectively, with all points lying essentially on the diagonal, suggesting that ATV and ITV across those 15 brain areas are essentially numerically identical. Intriguingly, during the evoked response (Fig. 3c), ATV for many brain areas is reduced compared to ITV. The amount of this reduction varies across areas, such that the regression coefficient between ATV and ITV across areas is reduced, yet still significant (*r* = 0.671, *p* = 0.006). Importantly, this reduction demonstrates that the ATV-ITV correlation coefficients close to one during the other periods are not trivial consequences of the data analysis, e.g., of deriving ATV and ITV from the same data.

### ATV is reduced below ITV when signal power is phase-aligned to an event

A closer inspection of the data points for individual areas revealed that the ATV reduction is particularly strong for early and intermediate-level visual areas V1, V2, and V4 (red dots in Fig. 3c), known to show relatively strong evoked responses to visual stimulus onset. By contrast, the ATV reduction is comparably weak for areas Tpt, S1, and F1 (green dots in Fig. 3c), which are not expected to show strong evoked responses to visual stimuli. Thus, ATV and ITV are almost identical in the absence of evoked responses, whereas during evoked responses, ATV is reduced compared to ITV, and this reduction is likely related to the evoked-response strength.

To further investigate the relationship between ATV reduction and the evoked-response strength, we assessed evoked-response strength by calculating the ratio of evoked power, i.e., the power of the signal components that are phase-locked to stimulus onset^36^, to total power. For the calculation of total power, power is calculated per trial and subsequently averaged over trials. By contrast, for the calculation of evoked power, the LFP is averaged over trials, resulting in the ERP, and subsequently, the power of this ERP is calculated. Evoked power can range between zero and the value of total power, such that the ratio of evoked to total power assesses the fraction of total power that is due to the ERP; we refer to this as the “evoked power ratio” (y-axis values in Fig. 3e–g). As expected, the evoked power ratio was large during the evoked-response period, ranging from 0.025 for areas Tpt, S1 and F1, to >0.3 for areas V1 and V2 (Fig. 3f). As also expected, the evoked power ratio was substantially smaller during the sustained response period (Fig. 3g), and even smaller during the pre-stimulus baseline (Fig. 3e). Importantly, the evoked power ratio was negatively correlated to the ratio of ATV over ITV for all three task periods. For the evoked-response period, this correlation assumed a value of 0.9996, suggesting that the ATV reduction (Fig. 3c) is most likely due to the ERP (Fig. 3f).

Given that most ATV studies have used human data, we extended our analysis to test the relationship between ATV and ITV using 64-electrode EEG data from 20 human subjects^37^. Both ATV and ITV were computed during four distinct periods of a working memory task (Fig. S1a; see “Methods” for details). For each of the four task periods, and each of the 64 electrodes, ATV and ITV were calculated. Within each task period, the correlation between ATV and ITV was assessed across 64 electrodes (Fig. S1b). The results consistently showed regression coefficients close to one (*r* > 0.99) and regression slopes (*s*) close to one, suggesting that ATV and ITV are essentially identical for human EEG data. The distribution of regression coefficients over all 20 healthy subjects indicated a similarly strong relationship between ITV and ATV for all subjects (Fig. S1c). Note that in these data, we did not observe clear reductions of ATV after an external event. Such an absence of ATV reduction could be due to weak phase resetting in this dataset due to stimuli variation across trials in their location, shape, and color.

### A simple model for the empirical relationships between ATV, ITV and ERPs

The above results in macaque ECoG data suggest that ATV is almost identical to ITV for periods without ERPs, whereas ATV is reduced relative to ITV for periods with ERPs. To further investigate this, we conducted a series of simple model simulations and also performed control analyses on example LFPs from the ECoG data (see “Methods”).

**Fig. 4:**
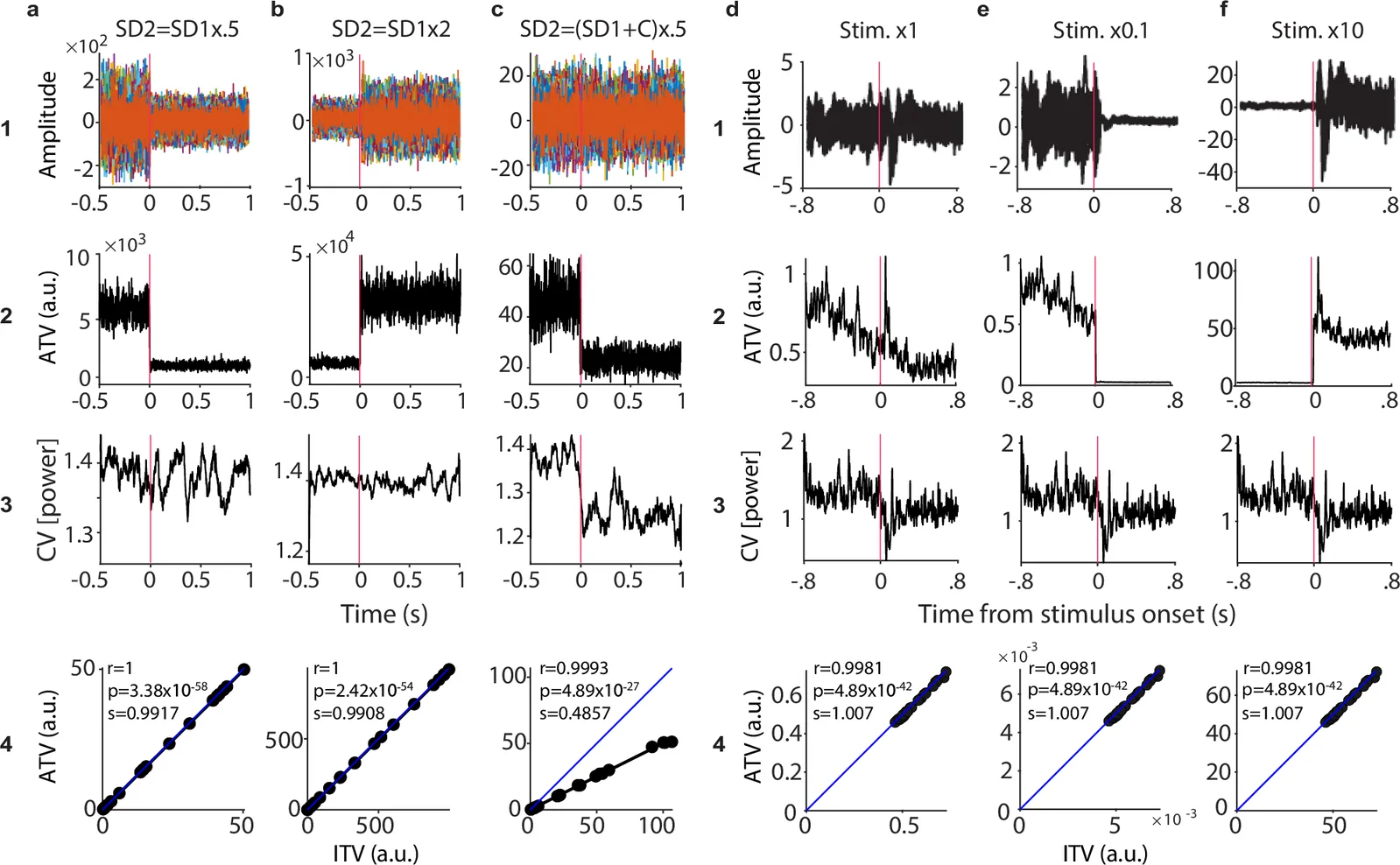
ATV change is driven by amplitude modulation or across-trial phase alignment, but CV(power) is power independent. **a–c** Simulated data. Each column shows 100 example simulated trials, drawn from two distributions, representing a fixation period (−0.5–0 s) and a stimulus period (0–1 s). The three columns model different conditions: **a** the stimulus period has half the SD compared to the fixation period, **b** the SD of the stimulus period was doubled compared to the fixation period, **c** before time zero, signals were sums of two independent white-noise processes varying across trials, whereas after time zero, one source was held constant across trials. The rows show: (1) the raw simulated signals, (2) the ATV, (3) the CV(power), (4) the correlation between ATV and ITV for the stimulus period. In row 4, each dot represents data from one of 20 separate simulations (simulated channels); the blue line indicates equality between ATV and ITV; the values in the upper right corners provide the Pearson correlation coefficients (*r*), its significance levels (*p*), and the slopes of a linear regressions (*s*, shown as black line), between ATV and ITV across the 20 simulations. **d–f** Empirical data. Same as (**a**–**c**), but for 150 example trials of ECoG data from monkey K during 0.8 s of fixation and 0.8 s stimulus presentation. Column (**d**) shows the data as they were recorded. Column (**e**) shows the data after scaling them by a factor of 0.1 during the stimulus period. Column (**f**) shows the data after scaling them by a factor of 10 during the stimulus period. In row 4, each dot represents data from one ECoG channel.

We first simulated 100 trials of normally distributed white noise, whose amplitude was either decreased (Fig. 4a1) or increased (Fig. 4b1) at time zero. These changes in white-noise amplitude constitute changes in wideband power, i.e., wideband ITV. As ITV was essentially identical to ATV in experimental data without ERPs, we expected the same to hold for those simulated data. Indeed, Fig. 4a2, b2 shows that a decrease or increase in intra-trial amplitude led to a decrease or increase in cross-trial ATV, respectively.

Next, we simulated an ERP that was generated by a partial phase reset at time zero^38^. A phase reset leads to a signal component that is event-locked across trials. We simulated this event-locked component as a white-noise process that was identical for all 100 trials. This event-locked component was not supposed to change the overall power, i.e., the ITV of the signal, which was accomplished as follows: The signal before time zero was generated as the superposition of two independent white-noise processes of equal mean and standard deviation (SD), which were also independent across trials. The signal after time zero was generated in the same way, except that one of the two white-noise processes was constant across trials. In other words, a constant time-varying ERP was simulated across trials. The results are shown in Fig. 4c1, c2: At time zero, the raw signals across the 100 trials show no apparent change (Fig. 4c1), yet the ATV was strongly reduced (Fig. 4c2). Intriguingly, the CV(power), our proposed metric for continuous neuronal signal variability, decreased during the stimulus period only in the third condition (Fig. 4c3)—where shared information existed across trials, and variability was expected to diminish—but not in the amplitude-change simulations (Fig. 4a3, b3).

We simulated 20 electrodes, each of which had an individual level of power, generated by an individual SD of the simulated white-noise process. This allowed us to analyze the relation between ATV and ITV in a similar way as we had done in Fig. 3b–d. For these analyses, we used the 1 s period after time zero. For the data without a simulated ERP (columns a and b), the regression coefficient between ATV and ITV, across the 20 electrodes, assumed the value of one, and the regression slope was also one; that is, ATV and ITV were identical (Fig. 4a4, b4). These simulation results were expected from a white-noise process, where each data point is drawn independently, such that variance calculated across time or trials is expected to be the same, and we show this mainly for illustration and as context for the next simple simulation. For the data with a simulated ERP (column c), the regression coefficient was 0.99, yet the regression slope was reduced to 0.496, that is, to a value very close to half (Fig. 4c4). This illustrates how a constant component across trials, accounting for half of the total signal energy, in the absence of changes in power, i.e., ITV, can lead to reductions in ATV.

Finally, to emphasize the robustness of the proposed variability metric and its independence from power, we performed a control analysis on the 150 example trials of the ECoG data (Fig. 4d1). We scaled the data during the stimulus period by dividing it by a factor of 10 (Fig. 4e1) or multiplying it by 10 (Fig. 4f1). The results demonstrate that the ATV of the LFP changes with scaling (Fig. 4d2–f2). By contrast, the CV(power) remains stable (Fig. 4d3–f3). Because the SD is the square root of variance, and variance scales with the squared signal, the SD scales proportionally with the mean. Consequently, the CV(power) remains constant under amplitude scaling, highlighting its suitability as a power-independent measure of variability. The regression coefficient between ATV and ITV, across channels, assumed values very close to one, and the regression slopes were also close to one; that is, ATV and ITV were essentially identical (Fig. 4d4). This relationship was not affected by the applied amplitude scaling (Fig. 4e4, f4).

### The mean-normalized metric for LFP variability

**Fig. 5:**
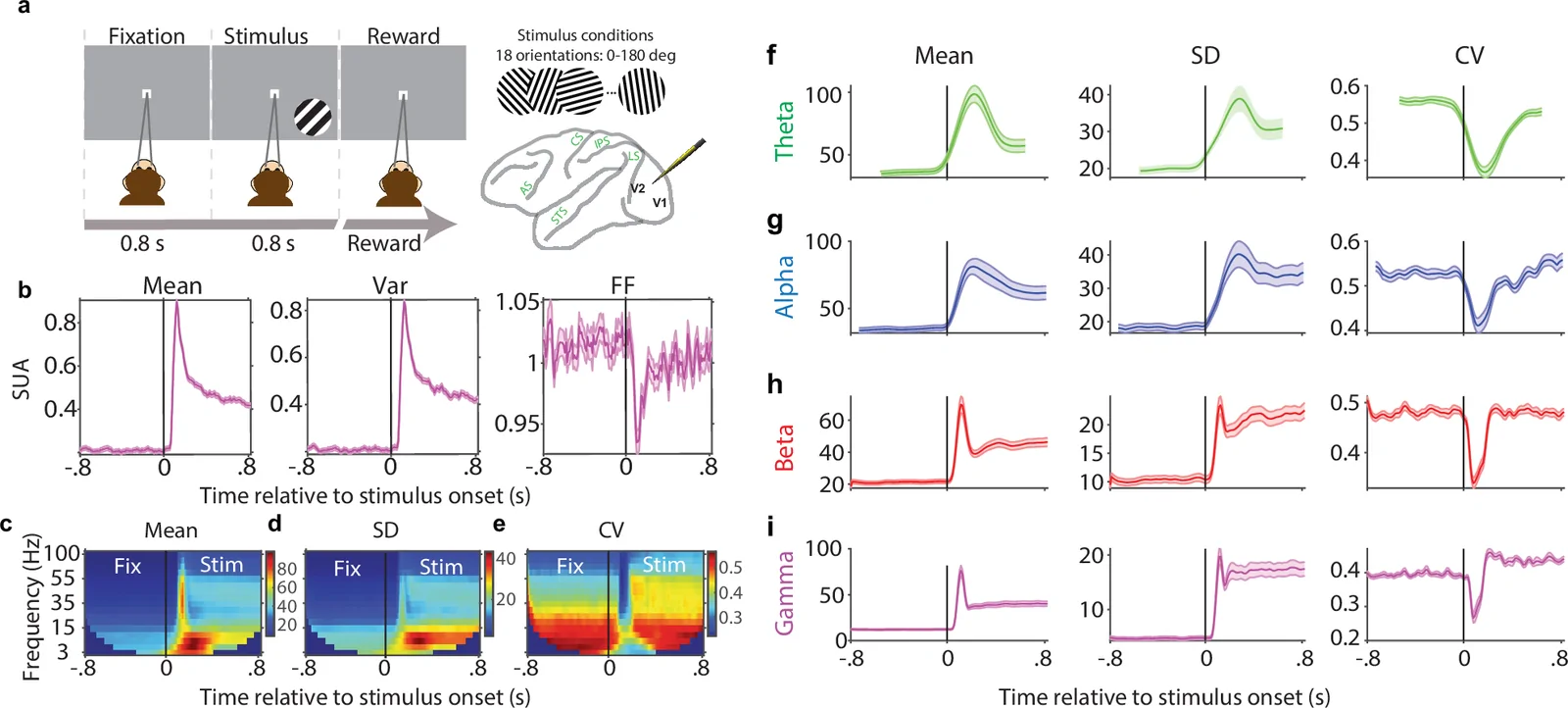
The CV(power) as a mean-normalized variance metric for LFP power. Data from intracortical microelectrode recordings, including single-unit recordings. **a** Schematic representation of the passive fixation task, during which microelectrode data were recorded. The schematic was designed using Adobe Illustrator, incorporating modified graphical elements originally published in ref. ^56^. **b** Spike-count mean (left), variance (middle), and Fano Factor (right), computed within 0.05 s sliding windows. Time-frequency representation of the mean (**c**), SD (**d**), and CV (**e**) of the power. Shown are the averages over 90 repetitions of a single stimulus. **f** Time courses of the mean, SD, and CV, averaged over the theta band to better illustrate the stimulus-related dynamics. Solid lines represent the group means, and shaded error bands denote ± one standard error of the mean (SEM) across sessions (*n* = 20). Same as (**f**), but for the alpha (**g**), beta (**h**), and gamma (**i**) bands.

Spike-count variability is reduced by stimulus onset, and it is an interesting question whether stimulus onsets, or other relevant events, also quench LFP power variability. Inspired by our simulation findings, we extended our investigation of CV(power) into the time-frequency domain by analyzing an LFP dataset (Fig. 5a) and comparing CV(power) of the LFP to the Fano Factor of the spike trains recorded simultaneously at the same sites.

We first calculated the single-unit SC Fano Factor in intracortical microelectrode recordings from awake macaque area V1 (Fig. 5b), and confirmed that it was reduced after stimulus onset (Fig. 5b, *N* = 232 single units recorded in 22 sessions in one macaque). We then calculated the LFP power, its SD, and its CV, and show those metrics as time-frequency plots (Fig. 5c–e) and as time courses for several frequency bands (Fig. 5f–i). Note that in this dataset, visual stimulation did not decrease low-frequency power but increased it. Crucially, CV(power) in the theta band (Fig. 5f) and alpha band (Fig. 5g) showed stimulus-related dynamics that were quite similar to those of the SC Fano Factor, entailing a stimulus-related transient decrease and a slow return. The CV(power) in the beta band (Fig. 5h) showed primarily the transient reduction. The CV(power) in the gamma band (Fig. 5i) showed a transient reduction followed by a sustained increase. Note that the different dynamics of CV(power) observed for the different frequency bands suggest distinct oscillatory phenomena rather than a spreading of dynamical activity across the spectrum due to a non-sinusoidal nature of LFP oscillations. Note further that the peak changes in CV(power) are troughs approximately 100 ms after stimulus onset, indicating a temporal reduction in “noise-to-signal” at a latency reminiscent of the classical P100.

**Fig. 6:**
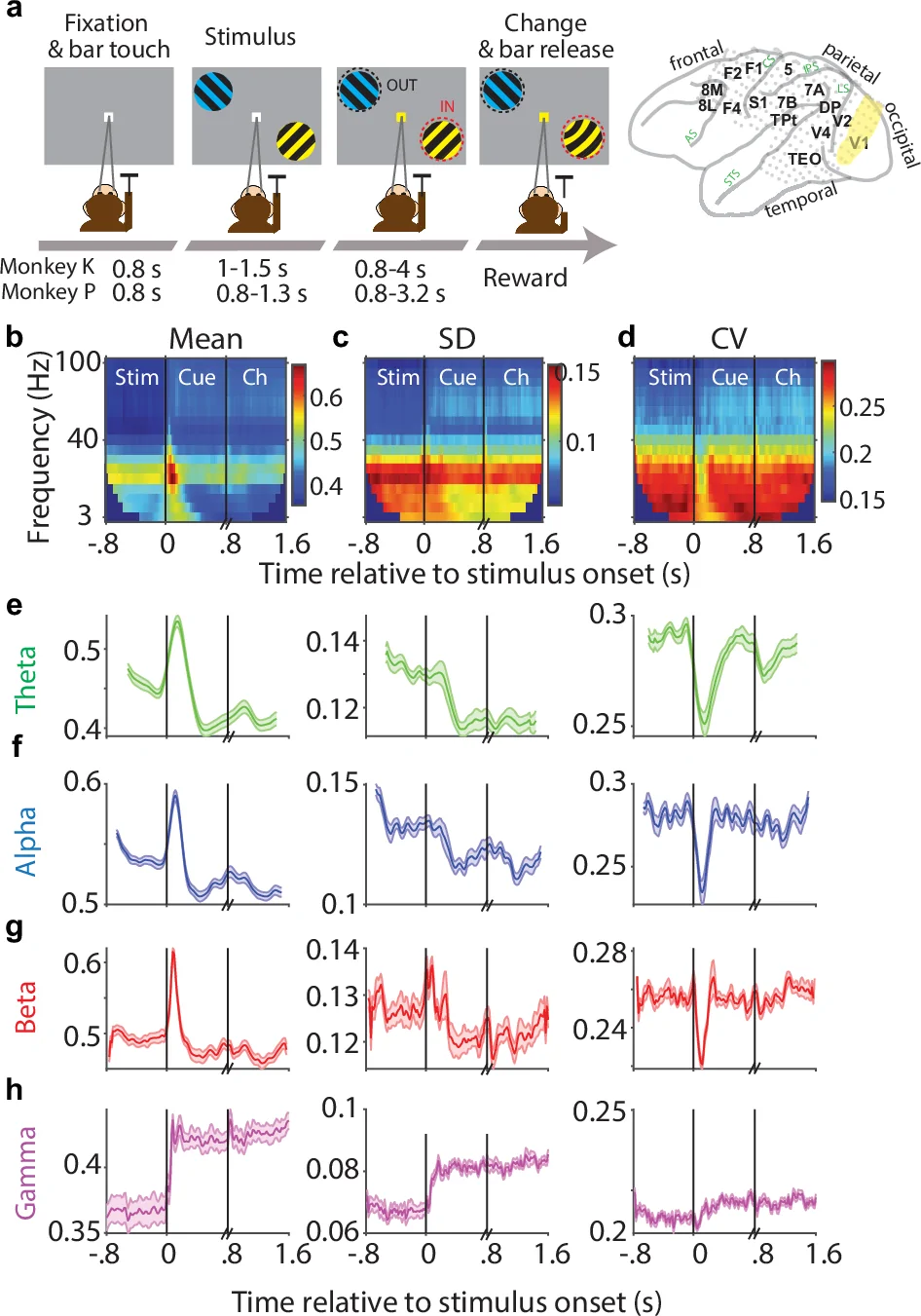
The CV(power) as a mean-normalized variance metric for LFP power. Data from subdural ECoG array (Monkey K). **a** Schematic illustration of the attention task, during which ECoG data were recorded. The analysis used the data from area V1, highlighted in yellow. AS Arcuate Sulcus, CS Central Sulcus, STS Superior Temporal Sulcus, IPS Intraparietal Sulcus, LS Lateral Sulcus. The schematic was designed by the authors using Adobe Illustrator, incorporating modified graphical elements originally published in refs. ^33,64^. Time-frequency representation of the mean (**b**), SD (**c**), and CV (**d**) of the power. Shown are the averages over 317 attend-IN trials. **e** Time courses of the mean, SD, and CV, averaged over the theta band to better illustrate the stimulus-related dynamics. Solid lines represent the group means, and shaded error bands denote ± one standard error of the mean (SEM) across 9 sessions times 2 stimulus conditions (*n* = 18). Same as (**e**), but for the alpha (**f**), beta (**g**), and gamma (**h**) bands.

Next, we investigated CV(power) for the V1 recording electrodes from the ECoG dataset (Fig. 6). CV(power) in the theta band showed transient reductions both after stimulus onset and after attentional cue onset (Fig. 6e). CV(power) in the alpha band (Fig. 6f) and the beta band (Fig. 6g) showed primarily stimulus-related transient reductions. CV(power) in the gamma band primarily showed a stimulus-related sustained increase. Implementing the same analysis for the data from a second monkey (Monkey P), whose dominant frequency peaks differed slightly (gamma: 55–75 Hz; alpha-beta: 6–30 Hz), yielded overall similar results (Fig. S2).

**Fig. 7:**
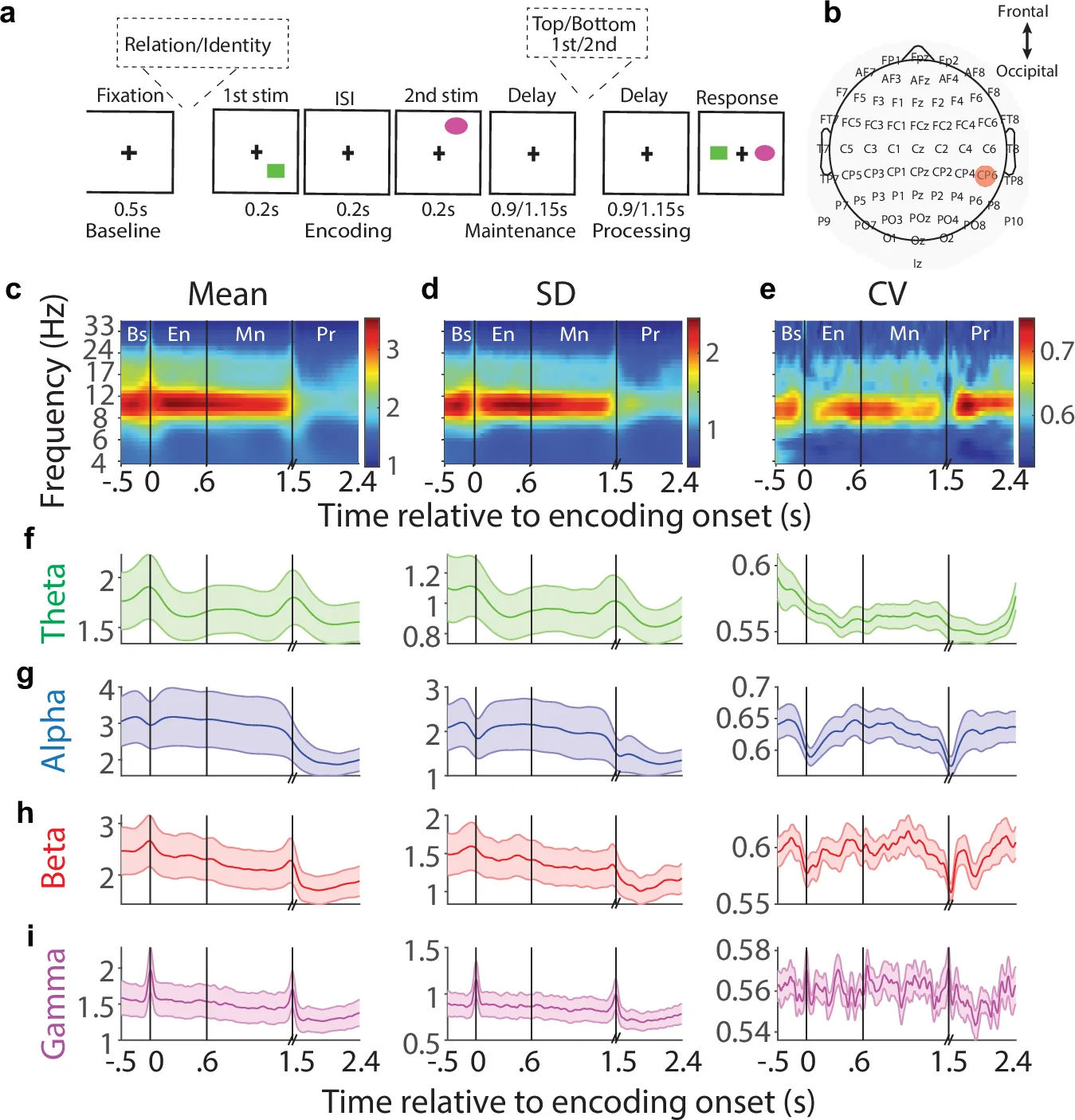
The CV(power) as a mean-normalized variance metric for LFP power. Data from human EEG recordings. **a** Schematic illustration of the working memory task, during which EEG data were recorded. **b** The analysis used the data from the centro-parietal electrode CP6, highlighted in red. Time-frequency representation of the mean (**c**), SD (**d**), and CV (**e**) of the power between 1 and 40 Hz. Shown are the averages over 20 subjects. Bs baseline, En encoding, Mn maintenance, and Pr active processing. **f** Time courses of the mean, SD, and CV, averaged over the theta band to better illustrate the stimulus-related dynamics. Solid lines represent the group means, and shaded error bands denote ± standard error of the mean (SEM) across subjects (*n* = 20). Same as (**f**), but for the alpha (**g**), beta (**h**), and gamma (**i**) bands.

We further calculated CV(power) for the EEG dataset (Fig. 7). The CP6 channel was selected for its task-induced power perturbations (Fig. 7b, c). As in previous analyses, we computed the mean (Fig. 7c), SD (Fig. 7d), and coefficient of variation (CV; Fig. 7e) of the power. CV(power) in the theta band (Fig. 7f) showed reductions relative to the baseline period during all task periods. CV(power) in the alpha band (Fig. 7g) and the beta band (Fig. 7h) showed primarily transient reductions related to the start of Encoding and Processing. CV(power) in the gamma band (Fig. 7i) showed small increases around the start of Encoding and Processing and some reduction during Processing.

## Discussion

We found that ATV, outside of ERPs, is essentially identical to ITV. This confirms predictions of mean-field neuronal-population models, in which the LFP is the result of a stochastically driven near-linear system^22–24^ and, outside of ERPs, is ergodic. The ATV-ITV correlation coefficients close to one were revealed by calculating the two metrics in strictly corresponding ways, by first filtering the data and then computing variance either across trials (ATV) or within trials (ITV). Importantly, ITV is equivalent to the commonly used spectral power. Thus, the strong correlation between ITV and ATV suggests that ATV reflects spectral power during periods devoid of ERPs. For periods with ERPs, ATV is reduced compared to ITV. The ratio of ATV over ITV is strongly negatively correlated with the ratio of evoked power over total power, a metric of the ERP’s contribution to the LFP. These effects were recapitulated in very simple simulations. Furthermore, we propose a metric for the mean-normalized variability of LFP power, namely the CV(power), and we show CV(power) for several datasets and task conditions.

The CV(power) metric provides a principled alternative to the non-normalized ATV. CV(power) aligns more closely with mean-normalized variability measures such as the Fano factor. It is also theoretically motivated by signal detection theory: the CV(power) corresponds to the inverse of d’ (i.e., discriminability) and is inversely correlated with expected performance when using signal power as a metric for decoding (as expected for a variability measure). By considering LFP power rather than LFP amplitude, and by normalizing LFP-power variability by its mean, CV(power) can be interpreted within a signal detection framework as an inverse proxy for discriminability, linking it more directly to population coding and decoding efficiency. This establishes a clearer bridge between electrophysiological observations and computational theories of neuronal variability. Note that it is well possible that in some cases, the cognitively or behaviorally most relevant LFP metric is LFP power itself rather than CV(power). Yet, if investigators aim at quantifying LFP variability, it is crucial to realize that the non-normalized ATV turns out to reflect LFP power (outside of ERPs). LFP power quantifies LFP variance, and it typically cannot be meaningfully normalized by the LFP mean, because the LFP mean outside of ERPs is zero (as the LFP and EEG are typically recorded after high-pass filtering, or “AC coupling”). By contrast, the LFP-power mean is always positive, just like the spike count, and therefore can provide a meaningful ground for a mean-normalized variability metric, which leads us to CV(power).

Previous studies have linked elevated ATV in E/MEG signals to impaired neuronal stability in conditions such as autism, ADHD, and schizophrenia^15,39–43^. Our findings suggest that such interpretations may need to be revisited, as changes in ATV could partly reflect alterations in power rather than intrinsic variability. In contrast, CV(power), as a mean-normalized measure, may provide a more appropriate marker for assessing neuronal variability in both cognitive and clinical contexts.

EEG/MEG/LFP signals are fundamentally different from neuronal SCs. While SCs reflect the output of neurons along their axons, EEG/MEG/LFP signals reflect the synaptic inputs on their dendrites and somata and the corresponding postsynaptic currents. The postsynaptic currents summate to the EEG/MEG/LFP when they are synchronized, and this synchronization is greatly aided by neuronal oscillations. Correspondingly, both changes in the degree of synchronization and in the number of postsynaptic currents can contribute to the physiological variability of EEG/MEG/LFP oscillations. Oscillations render the spike outputs of the respective neurons, and the resulting post-synaptic currents at their target neurons partly temporally predictable, and thereby provide a natural way for oscillatory synchronization. Oscillatory synchronization occurs at characteristic frequency bands, and oscillations of various frequencies overlap to form the LFP, EEG and MEG. These oscillations have been linked to neuronal communication and processing^44,45^.

Neuronal responses, and also ensuing behavioral responses, to a given input can be modulated depending on the oscillation phase. However, crucial for the present study, the momentary absolute phase of an ongoing oscillation does not seem to be a code that can be directly compared to the neuronal SC code. Let us assume that a particular stimulus induces a pattern of SCs across a group of neurons. Then, by recording this pattern of SCs for a short window of time, one can decode the stimulus with high fidelity. Let us now assume that these neurons engage in an ongoing oscillation. Then, by recording and estimating the instantaneous oscillation phase, one probably cannot decode the stimulus. The phase undergoes a full rotation during each oscillation cycle, such that it does not differentiate between stimuli as long as all stimuli induce the oscillation to some degree. Note that the stimulus might well be decoded from the pattern of phase relations between recording sites^46,47^ or from the pattern of oscillation strengths^48–50^. This is different from the instantaneous oscillation phase of one particular recording site. Correspondingly, the phase of an ongoing neuronal oscillation at a particular time point typically varies randomly across trials, except when the oscillation is transiently reset, e.g., by a stimulus onset or a (micro)saccade. These resets can lead to evoked responses^38^, during which the phase and the corresponding amplitude of the LFP signal do carry information about the respective event. Yet, neuronal oscillations are not sinusoidal oscillations with linear phase evolution, but rather their phases drift substantially (which also contributes to them typically occupying a spectral band rather than a spectral line). Thereby, after a resetting event, the oscillation phase drifts, and after a few cycles is again random across trials. As the oscillation phase directly maps onto the raw instantaneous signal amplitude, this instantaneous amplitude value is equally random across trials. From these considerations, we suggest that the instantaneous amplitude of the ongoing LFP, EEG, or MEG signal, outside of evoked responses, is not suited for stimulus decoding in the same way as neuronal SCs are. Correspondingly, while the ATV of SCs is highly meaningful, the meaning of the ATV of the instantaneous EEG amplitude is less clear.

Here, we have shown empirically that the ATV of the LFP (or EEG) amplitude is very highly correlated with the ITV of LFP (or EEG), that is to power, a standard metric derived from the LFP, EEG, or MEG. This identity holds for periods outside of evoked responses. During evoked responses, the strength of the evoked response determines how much ATV is reduced relative to the ITV. Because stimulation does induce reliable changes in LFP power, we investigated whether the variability of LFP power, rather than the raw LFP amplitude, showed interesting effects of stimulation. Indeed, we found that CV(power) is a suitable metric. Intriguingly, this metric is constructed similarly to the Fano Factor of single-unit SCs. To our knowledge, the CV(power) has so far not been systematically studied as a function of stimuli and tasks^51,52^. We envisage that many such studies can easily be performed on existing and future LFP, EEG, and MEG datasets. These studies would thereby investigate whether and how these continuous neuronal signals show similar variability quenching as has been prominently described for single-unit SCs^4^.

## Materials and methods

We calculated ATV and ITV on an ECoG dataset from one awake macaque, on a microelectrode dataset from one awake macaque, on an EEG dataset from human subjects, and on simulated data. Additionally, we quantified SC variability using the Fano Factor for the microelectrode dataset, and we quantified LFP power variability by the coefficient of variation for the microelectrode, the ECoG, and the EEG dataset.

### Ethics

All empirical datasets have been acquired under respective ethical approvals. For the ECoG dataset, collected from two male macaque monkeys, all procedures were approved by the animal ethics committee of Radboud University Nijmegen (approval number: RU-DEC-2004-151). For the intracortical microelectrode dataset, electrophysiological recordings were performed in a single awake male macaque monkey. All surgical procedures and housing conditions were compliant with German national animal welfare regulations and European Union guidelines for the care and use of laboratory animals (EU Directive 2010/63/EU). All experimental procedures were approved by the regional authority, Regierungspräsidium Darmstadt (approval number: F149/1008). For the human EEG data, written informed consent was obtained in accordance with the Declaration of Helsinki, formally reviewed and approved by the University of California, Berkeley, Institutional Review Board (reference number: 2010-02-783) or the Regional Committee for Medical Research Ethics, Region South (reference number: 2014/381). All datasets have been used in previous publications, which describe the experiments in detail^30,33,34,37,53–56^. The following methods focus on the specific data analyses for the current study.

### Datasets

#### ECoG dataset

In the ECoG dataset (Figs. 1–4 and 6), local field potentials were recorded using an ECoG electrode grid (1 mm electrode diameter and 2–3 mm electrode spacing) implanted on the left hemisphere of two macaque monkeys (Monkey K and Monkey P)^30^. LFPs were recorded using Plexon headstages and a Neuralynx data acquisition system. Signals were filtered between 0.159 Hz and 8 kHz, digitized at ~32 kHz, low-pass filtered at 250 Hz, and downsampled to 1 kHz. For the current study, we used data from both monkeys. Non-human primate research customarily involves only two or three individuals, and statistical inferences in this context are accordingly limited to those subjects rather than to the population^57,58^. The monkeys were trained to perform a selective visual attention task, requiring them to maintain their gaze on a central fixation point and press a lever throughout a trial. After a 0.8 s fixation period, two patches of sinusoidal grating appeared, drifting behind a circular aperture, one colored blue, the other yellow, with the colors randomly distributed across trials. A subsequent change in the fixation point’s color cued the monkeys to attend to the stimulus with the matching color. The monkeys had to release the lever in response to a shape change in the attended stimulus to obtain a reward, while ignoring changes in the non-attended stimulus. Monkey K achieved 94% accuracy, monkey P 84%. During the task, LFPs were recorded from 252 channels across 15 brain areas.

We have previously established that the two most prominent brain rhythms in these data are the gamma rhythm in the occipital region (defined here as areas V1, V2, V4) and the beta rhythm in the frontal region (defined here as areas F1, F2, F4)^33^. Correspondingly, the current analysis focused on those rhythms and regions. Note that the frequency-band definitions in the present study differed slightly from those in the previous study. Trials with attention directed to the visual hemifield contralateral to the recorded hemisphere were referred to as the attend-IN (or just IN) condition, and trials with attention ipsilateral as the attend-OUT (or just OUT) condition. We considered only trials with at least 0.8 s stimulus presentation before the cue and 0.8 s between the cue and the stimulus change.

For Figs. 1, 2, 4, and 6, we segmented the data as follows: (1) “fixation” period: last 0.8 s of fixation end-aligned to stimulus onset; (2) “stimulus” period: first 0.8 s after stimulus onset; (3) “cue” period: first 0.8 s after cue onset; (4) “change” period: last 0.6 s end-aligned to stimulus change. For Fig. 3, we segmented the data differently because we aimed at contrasting periods with and without ERP: (1) “fixation” period: last 0.5 s of fixation end-aligned to stimulus onset; (2) “ERP” period: first 0.3 s after stimulus onset; (3) “sustained” period: the 0.5 s between 0.3 and 0.8 s after stimulus onset.

#### Microelectrode data

In the microelectrode dataset (Fig. 5), LFP and single-unit spike data were intracortically recorded from areas V1 and V2 of a macaque monkey during a fixation task. Recordings were made using Plexon V-probes with 24 or 32 contacts spaced 100–150 μm apart. The raw data were initially recorded at a sampling rate of 24,414.0625 Hz using Tucker Davis Technologies systems and subsequently downsampled offline to 1 kHz. The monkey maintained central fixation throughout each successfully completed trial and was rewarded for this with grape juice. Following a 1–1.1 s baseline period, the monkey was presented with a grating stimulus for 1–1.3 s. Grating orientations varied from 0° to 170° in 10° steps, with one orientation presented per trial. Each recording session consisted of three stimulus blocks. In the first and third blocks, all orientations were presented in a pseudorandom sequence. In the second block, one specific orientation (out of 18 orientations) was randomly selected for each recording session and was presented 90 times in a row. We analyzed the 90 consecutive trials of the same stimulus from the second block. Spike data were sorted into single units using SpyKING CIRCUS^59^.

#### EEG data

The EEG dataset (Fig. 7) was collected from two groups of subjects: 14 individuals with unilateral prefrontal cortex lesions (lesion group) and 20 age- and education-matched healthy controls (control group). EEG signals were recorded from 64 + 8 channels using a BioSemi ActiveTwo amplifier, with Ag-AgCl electrodes positioned according to the 10–20 system, sampled at 1024 Hz^37^. Data were downsampled to 256 Hz for the main analyses. For the current study, we analyzed the EEG data from the 20 healthy human subjects in the control group (44 ± 19 [19–70] years of age, 16 ± 3 years of education, 11 males). Subjects completed a working memory task consisting of four phases: (1) baseline, (2) encoding, (3) maintenance, and (4) active processing. Each trial began with a 2-second fixation, followed by two sequential stimuli (0.2 s each) with a 0.2 s inter-stimulus interval. After a brief blank period (duration randomly either 0.9 or 1.15 s), subjects were asked to identify the relation (TOP/BOTTOM/FIRST/SECOND) or identity (SAME/DIFFERENT) between the two previously presented stimuli, followed by another delay period before responding by selecting the correct stimulus.

#### Simulated data

The simulation (Fig. 4) was designed to model ATV in a time series containing two distinct temporal segments with different variability values. Three different conditions were implemented to manipulate the amplitude and structure of variability in these two periods, and the relationship between ATV and ITV was subsequently quantified via correlation analysis.

The simulated data were drawn from two distributions, representing a fixation period (0.5 s) and a stimulus period (1 s). The distributions were standard normal distributions according to the following formula:

$$R\left(x\right)=A\times \tfrac{1}{\sqrt{2\pi }}{e}^{-\frac{{x}^{2}}{2}}$$

where *x* is a random variable with a mean of 0 and variance of 1, and *A* represents the standard deviation of the distribution.

The simulated dataset was generated with a sampling frequency of 1000 Hz, resulting in 1500 samples per trial over a 1.5 s trial duration. Each condition contained 20 simulated subjects, each performing 100 trials. For all conditions, the first segment was generated with Gaussian noise scaled by a subject-specific random factor. The second segment included condition-specific scaling and, for some conditions, a proportion of a “fixed term”. The three different scenarios were modeled as follows: (1) the stimulus period has half the SD compared to the fixation period; (2) The stimulus period has double the SD compared to the fixation period; (3) The signal before time zero was generated as the superposition of two independent white-noise processes of equal mean and SD, which were also independent across trials. The signal after time zero was generated equally, except that one of the two white-noise processes was constant across trials. The situation after time zero models a time-varying ERP generated by phase-alignment of half of the signal energy across trials. Pearson’s correlation coefficients were calculated between ATV and ITV across simulated subjects for each condition.

### Analysis

All analyses were performed using MATLAB 2020b utilizing the FieldTrip toolbox^60^. Line-noise artifacts were removed for the ECoG data at 50 Hz and harmonics and for the EEG data at 60 Hz and harmonics. For the microelectrode data, line-noise removal was not necessary. For all LFP and EEG signals, we calculated one mean value and one SD value per channel (across all data periods used for analysis), and used those values to normalize all values of the corresponding channel (z-transformation by subtraction of the mean and division by the SD). For this, the respective data were first segmented into epochs and cleaned of artifacts, and subsequently, mean and SD values were calculated across all data points pooled over all valid epochs, separately per electrode or recording site. ATV was first calculated from wideband data: For ECoG and microelectrode data, signals were filtered between 1 and 100 Hz, for EEG data, signals were filtered between 1 and 40 Hz. Subsequently, band-pass filtering was applied using the *ft_preprocessing* function in FieldTrip. For each frequency band of interest, a zero-phase two-pass Butterworth IIR band-pass filter was applied to each trial, using FieldTrip’s default filter settings.

ATV was estimated based on across-trial variance for each 1 ms time bin, and smoothed using a 0.005 s sliding window. ATV was calculated as follows:

$${ATV}(t)=\frac{1}{N}{\sum}_{i=1}^{N}{({x}_{i}(t)-\overline{x}(t))}^{2}$$

where ${x}_{i}(t)$ is the amplitude of the signal at time *t* in trial *i*, ${\bar{x}}_{t}$ is the mean amplitude over trials at time t, and N is the number of trials. Note that the equation we use to define ATV is mathematically similar to the variance-based approach used previously for calculating band-limited event-related synchronization or desynchronization^61^.

ITV was calculated as follows:

$${{ITV}}_{i}=\frac{1}{T}{\sum}_{t=1}^{T}{({x}_{i}(t)-{\overline{x}}_{i})}^{2}$$

where ${x}_{i}(t)$ is the amplitude of the signal at time *t* of trial *i*, ${\bar{x}}_{i}$ is the mean amplitude within trial *i*, and T is the number of time points.

In the ECoG dataset, both ATV and ITV were calculated separately for each of 15 brain areas, and the Pearson regression coefficient between ATV and ITV across brain areas was assessed for different periods: (1) the fixation period (last 0.5 s before stimulus onset), (2) the evoked response period (first 0.3 s following stimulus onset), and (3) the sustained-response period (0.3 to 0.8 s after stimulus onset). ITV was calculated per trial and per period across the time points, and then averaged over trials. ATV was calculated across trials per time point, and then averaged over time points. Both ITV and ATV were calculated for individual recording channels and subsequently averaged over channels within each recording area, separately for the three periods.

We calculated the power as the squared absolute value of the signal. For power-spectrum analysis, the signals were multiplied by a single Hann taper, zero-padded to 1 s, and Fourier transformed. Power spectra were computed as the squared magnitude of the Fourier transform. Power spectra were calculated using the *ft_freqanalysis* function in the FieldTrip toolbox. Because the timing of the stimulus change was jittered and variable across trials, power spectra in Fig. 2 were computed for two distinct epochs within the attention period: (1) the start-aligned window, which spans the first 0.8 s following cue onset, and (2) the end-aligned window, which captures the final 0.6 s preceding the stimulus change. The power was calculated in the last 0.5 s of these periods.

The Fano factor is defined as the ratio of the variance to the mean of spike counts across trials within a given time window:

$${{\mathrm{Fano}}}\,{{\mathrm{factor}}}=\frac{{\sigma }^{2}}{\mu }$$

where ${\sigma }^{2}$ and μ are the variance and mean, respectively, of spike counts across trials within a given time window. Here, the Fano factor was computed using sliding windows of 0.05 s with 0.01 s steps.

Similarly, the CV(power) is defined for continuous neuronal signals as:

$${CV}({power})=\frac{\sigma }{\mu }$$

where σ and μ are the SD and mean, respectively, of power across trials, computed within a given time-frequency window.

Both measures normalize variability by the mean, thereby controlling for changes in variability that are directly proportional to changes in mean activity level. The key distinction is that the Fano factor normalizes variance by the mean, which is appropriate for count data that follow Poisson-like statistics, whereas CV(power) normalizes the SD by the mean, which is appropriate for continuous, amplitude-scaled signals.

### Statistics and reproducibility

Statistical tests were two-sided. Non-parametric Wilcoxon rank-sum tests were used for group comparisons. For figure 2, ATV was computed using 100 repeated random subsamplings of trials within each session, and power was calculated at the single-trial level. The resulting distributions of ATV or power values were compared either between task epochs (stimulus/cue vs. fixation) or between IN and OUT conditions, using pooled data across sessions where applicable. Relationships between variables were assessed using Pearson correlation and linear regression. Analyses were conducted on repeated measurements across trials and recording sites within each dataset. No formal tests of normality were performed, and no correction for multiple comparisons was applied. Sample sizes (*n*) correspond to the number of trials, channels, or subjects as specified in each analysis. Variability measures are reported directly as defined in the text (e.g., ATV, CV(power)).

### Reporting summary

Further information on research design is available in the Nature Portfolio Reporting Summary linked to this article.

## Supplementary information

## Data Availability

The data underlying the main figures are publicly available on Zenodo [https://doi.org/10.5281/zenodo.21946658] and can be cited as^62^. The human EEG data analyzed in the current study were obtained from a previously published repository at [https://crcns.org/data-sets/pfc/pfc-5/about-pfc-5]. These datasets were originally generated and described in previous publications^30,37,56^.

## Code availability

The scripts for generating simulations and for analyzing neuronal and simulated data using MATLAB 2020b are publicly available on Zenodo [https://doi.org/10.5281/zenodo.21959626] and can be cited as^63^.
