# Supplementary material for: On variability in local field potentials
Source: Commun Biol. 2026 Sep 2;9:1165. doi: 10.1038/s42003-026-10881-x (PMC13538653; doi:10.1038/s42003-026-10881-x)
Supplement: Supplementary file 1 [file 42003_2026_10881_MOESM1_ESM.pdf]

## Supplementary information

# On variability in local field potentials

*Mohsen Parto-Dezfouli, Elizabeth L. Johnson, Eleni Psarou, Conrado Arturo Bosman, B. Suresh Krishna, Pascal Fries*

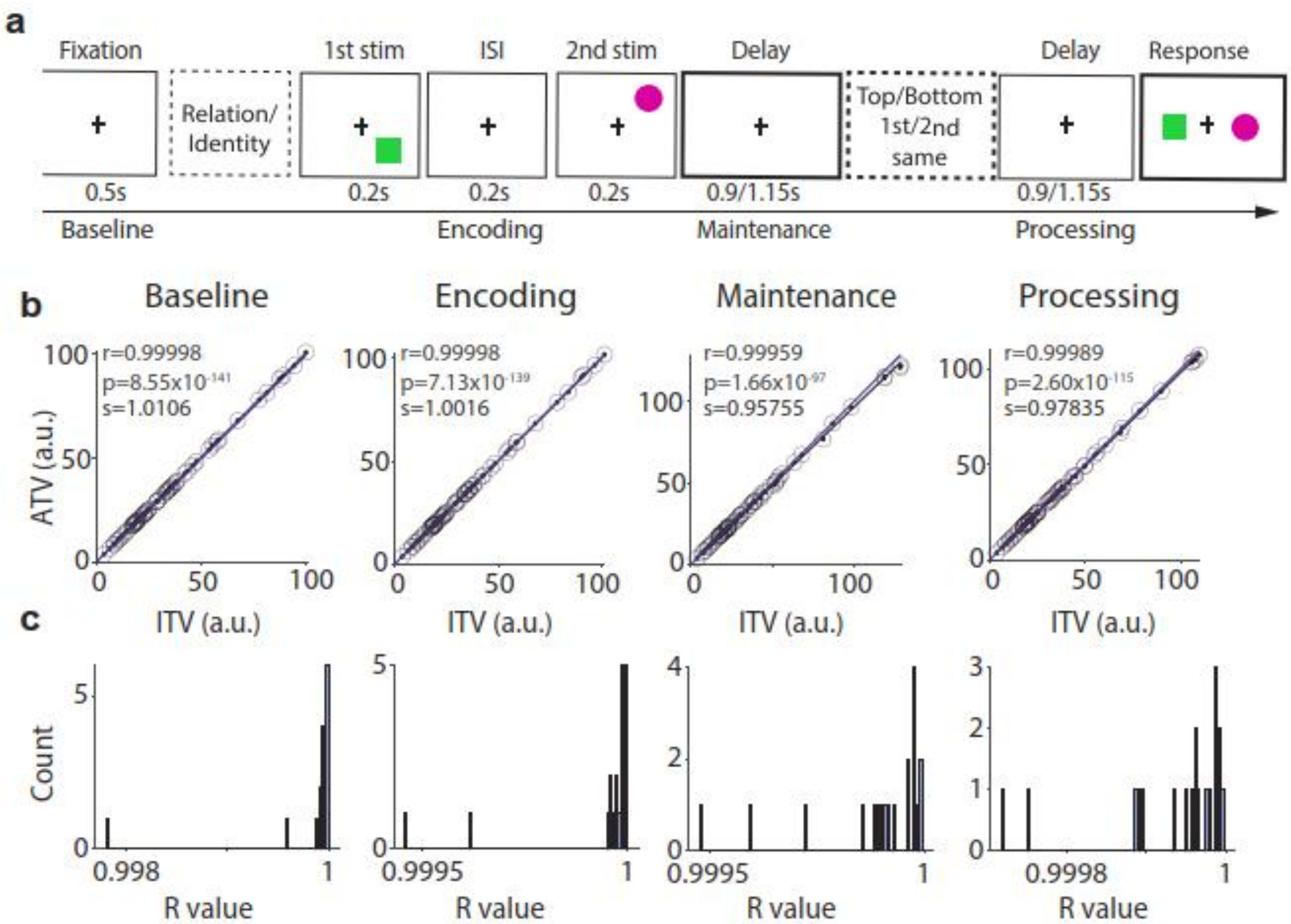


**Figure S1. ATV correlates with ITV for EEG data.**

**a**) Schematic illustration of the working memory task.

**b**) Correlation between ATV and ITV during the four task periods for an example subject. Each dot represents data from one recording channel.

**c**) Distribution of *r* values over all 20 healthy subjects.

# Supplementary information

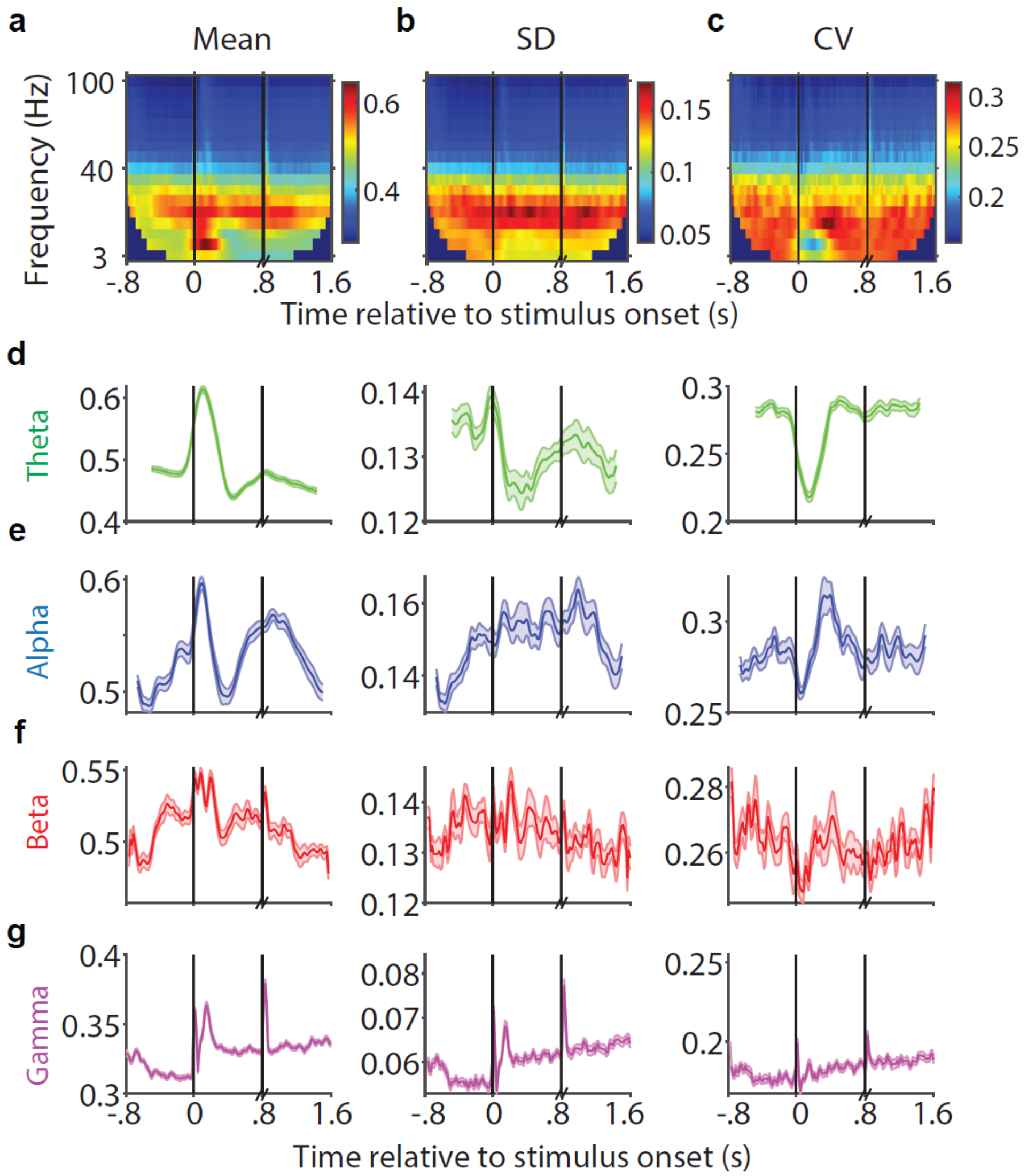


**Figure S2. The CV(power) as a mean-normalized variance metric for LFP power. Data from subdural ECoG array (Monkey P).**
**a-c**) Time-frequency representation of the mean (**a**), SD (**b**), and CV (**c**) of the power.
**d**) Time courses of the mean, SD, and CV, averaged over the theta band to better illustrate the stimulus-related dynamics. Solid lines represent the group mean, and shaded error bands denote ± one standard error of the mean (SEM) across 14 sessions times 2 stimulus conditions (n = 28).
**e-g**) Same as (**d**), but for the alpha (**e**), beta (**f**) and gamma (**g**) bands.
