# Supplementary material for: On variability in local field potentials
Source: Commun Biol. 2026 Sep 2;9:1165. doi: 10.1038/s42003-026-10881-x (PMC13538653; doi:10.1038/s42003-026-10881-x)
Supplement: Supplementary file 2 [file 42003_2026_10881_MOESM2_ESM.pdf]

nature portfolio

Corresponding author(s): Mohsen Parto-Dezfouli

Last updated by author(s): 16/08/2026

Reporting Summary

Nature Portfolio wishes to improve the reproducibility of the work that we publish. This form provides structure for consistency and transparency in reporting. For further information on Nature Portfolio policies, see our Editorial Policies and the Editorial Policy Checklist.

Statistics

For all statistical analyses, confirm that the following items are present in the figure legend, table legend, main text, or Methods section.

n/a	Confirmed	
☐	☒	The exact sample size (*n*) for each experimental group/condition, given as a discrete number and unit of measurement
☐	☒	A statement on whether measurements were taken from distinct samples or whether the same sample was measured repeatedly
☐	☒	The statistical test(s) used AND whether they are one- or two-sided<br>*Only common tests should be described solely by name; describe more complex techniques in the Methods section.*
☒	☐	A description of all covariates tested
☐	☒	A description of any assumptions or corrections, such as tests of normality and adjustment for multiple comparisons
☐	☒	A full description of the statistical parameters including central tendency (e.g. means) or other basic estimates (e.g. regression coefficient) AND variation (e.g. standard deviation) or associated estimates of uncertainty (e.g. confidence intervals)
☐	☒	For null hypothesis testing, the test statistic (e.g. *F*, *t*, *r*) with confidence intervals, effect sizes, degrees of freedom and *P* value noted<br>*Give P values as exact values whenever suitable.*
☒	☐	For Bayesian analysis, information on the choice of priors and Markov chain Monte Carlo settings
☒	☐	For hierarchical and complex designs, identification of the appropriate level for tests and full reporting of outcomes
☐	☒	Estimates of effect sizes (e.g. Cohen's *d*, Pearson's *r*), indicating how they were calculated

Our web collection on statistics for biologists contains articles on many of the points above.

Software and code

Policy information about availability of computer code

Data collection	Data were collected using previously published experimental setups; details are provided in the original studies referenced in the Methods (Datasets section). This study is conducted on pre-existing datasets and focused on methodological evaluation not a new cognitive signature.
Data analysis	Data were analyzed using MATLAB (R2020b) and the FieldTrip toolbox, along with custom scripts implemented by the authors. "The scripts for generating simulations and for analyzing neuronal and simulated data using MATLAB 2020b are publicly available on Zenodo [doi.org/10.5281/zenodo.21959626] and can be cited as 63 "

For manuscripts utilizing custom algorithms or software that are central to the research but not yet described in published literature, software must be made available to editors and reviewers. We strongly encourage code deposition in a community repository (e.g. GitHub). See the Nature Portfolio guidelines for submitting code & software for further information.

Data

Policy information about availability of data

All manuscripts must include a data availability statement. This statement should provide the following information, where applicable:

- Accession codes, unique identifiers, or web links for publicly available datasets
- A description of any restrictions on data availability
- For clinical datasets or third party data, please ensure that the statement adheres to our policy

The MATLAB scripts for generating simulations and analyzing neural data are available on GitHub, and the neural datasets are accessible via Zenodo. "The data

underlying the main figures are publicly available on Zenodo [doi.org/10.5281/zenodo.21946658] and can be cited as 62. The human EEG data analyzed in the current study were obtained from a previously published repository at [https://crcns.org/data-sets/pfc/pfc-5/about-pfc-5]. These datasets were originally generated and described in previous publications 30, 37, 56."

Research involving human participants, their data, or biological material

Policy information about studies with human participants or human data. See also policy information about sex, gender (identity/presentation), and sexual orientation and race, ethnicity and racism.

Reporting on sex and gender	Human EEG data were obtained from previously published datasets. we used only the "healthy adult" participants data for the current study. Demographic details are described in the original study: DOI: 10.1016/j.cub.2017.05.046. "We report data from 14 human adult patients with PFC lesions (mean ± SD [range]: 46 ± 16 [20–71] years of age, 15 ± 3 years of education, 5 males) and 20 age- and education-matched, healthy controls (44 ± 19 [19–70] years of age, 16 ± 3 years of education, 11 males)."
Reporting on race, ethnicity, or other socially relevant groupings	This paper is a method focus paper referred to original studies of the used human EEG and animal electrophysiological recording datasets.
Population characteristics	N.A.
Recruitment	Participants were recruited as part of previously published studies; recruitment procedures are described in the original publications.
Ethics oversight	For the human EEG data, written informed consent was obtained in accordance with the Declaration of Helsinki, formally reviewed and approved by the University of California, Berkeley, Institutional Review Board (reference number: 2010-02-783) or the Regional Committee for Medical Research Ethics, Region South (reference number: 2014/381).

Note that full information on the approval of the study protocol must also be provided in the manuscript.

Field-specific reporting

Please select the one below that is the best fit for your research. If you are not sure, read the appropriate sections before making your selection.

☒ Life sciences ☐ Behavioural & social sciences ☐ Ecological, evolutionary & environmental sciences

For a reference copy of the document with all sections, see nature.com/documents/nr-reporting-summary-flat.pdf

Life sciences study design

All studies must disclose on these points even when the disclosure is negative.

Sample size	20 healthy human subjects
Data exclusions	No additional exclusions beyond those defined in the original datasets were applied.
Replication	Analyses were performed on established datasets using standard and openly available methods; all findings were reproducible across datasets. The data underlying the main figures are publicly available on Zenodo.org.
Randomization	Not applicable, as this study is based on re-analysis of existing datasets and does not involve experimental group allocation.
Blinding	Blinding was not applied, as analyses were conducted on pre-existing datasets and focused on methodological evaluation.

Reporting for specific materials, systems and methods

We require information from authors about some types of materials, experimental systems and methods used in many studies. Here, indicate whether each material, system or method listed is relevant to your study. If you are not sure if a list item applies to your research, read the appropriate section before selecting a response.

Materials & experimental systems

n/a	Involved in the study
☒	☐ Antibodies
☒	☐ Eukaryotic cell lines
☒	☐ Palaeontology and archaeology
☐	☒ Animals and other organisms
☒	☐ Clinical data
☒	☐ Dual use research of concern
☒	☐ Plants

Methods

n/a	Involved in the study
☒	☐ ChIP-seq
☒	☐ Flow cytometry
☒	☐ MRI-based neuroimaging

Animals and other research organisms

Policy information about studies involving animals; ARRIVE guidelines recommended for reporting animal research, and Sex and Gender in Research

Laboratory animals	The study includes data from macaque monkeys; species and experimental details are reported in the original studies.
Wild animals	Laboratory animals
Reporting on sex	Male macaque monkeys.
Field-collected samples	All datasets have been used in sevral previous publications, which describe the experiments in detail.
Ethics oversight	All empirical datasets have been acquired under respective ethical approvals. For the ECoG dataset, collected from two macaque monkeys, all procedures were approved by the animal ethics committee of Radboud University Nijmegen (approval number: RU-DEC-2004-151). For the intracortical microelectrode dataset, electrophysiological recordings were performed in a single awake male macaque monkey. All surgical procedures and housing conditions were compliant with German national animal welfare regulations and European Union guidelines for the care and use of laboratory animals (EU Directive 2010/63/EU). All experimental procedures were approved by the regional authority, Regierungspräsidium Darmstadt (approval number: F149/1008).

Note that full information on the approval of the study protocol must also be provided in the manuscript.

Plants

Seed stocks	N.A.
Novel plant genotypes	N.A.
Authentication	N.A.
